# Force Plate Gait Analysis in Dogs After Femoral Head and Neck Excision

**DOI:** 10.3390/vetsci12050469

**Published:** 2025-05-14

**Authors:** Pongsatorn Tuchpramuk, Duangdaun Kaenkangploo, Thanikul Srithunyarat, Suvaluk Seesupa, Somphong Hoisang, Benedict Duncan X. Lascelles, Naruepon Kampa

**Affiliations:** 1 Faculty of Veterinary Sciences, Mahasarakham University, Mahasarakham 44000, Thailand; pongsathorn.t@msu.ac.th; 2 Division of Surgery, Faculty of Veterinary Medicine, Khon Kaen University, Khon Kaen 40002, Thailand; duakae@kku.ac.th (D.K.); thanikul@kku.ac.th (T.S.); suvalukse@kku.ac.th (S.S.); 3 Veterinary Teaching Hospital, Faculty of Veterinary Medicine, Khon Kaen University, Khon Kaen 40002, Thailand; sompho@kku.ac.th; 4 Translational Research in Pain Program, Comparative Pain Research and Education Centre, Department of Clinical Sciences, College of Veterinary Medicine, North Carolina State University, Raleigh, NC 27695, USA; dxlascel@ncsu.edu

**Keywords:** femoral head and neck excision, hip luxation, peak vertical force, symmetry index

## Abstract

Femoral head and neck excision (FHNE) remains a common orthopedic surgical procedure in many countries. However, data on postoperative outcomes regarding changes in hindlimb function are limited. This study aimed to evaluate hindlimb function after FHNE in dogs of different weights. Twenty-seven dogs that underwent FHNE were block-randomly assigned to two groups based on weight (≤20 kg and >20 kg). Hindlimb function was evaluated using force plate gait analysis to measure peak vertical force (PVF), alongside other orthopedic evaluations and a composite orthopedic assessment score at 1, 2, 3, 4, 5, 6, 9, and 12 months after surgery. Kaplan–Meier survival analysis and the log-rank test were used for statistical comparisons. The findings demonstrated no significant difference in PVF between the operated and non-operated limbs in either weight groups (≤20 kg and >20 kg) at four and three months postoperatively (median functional recovery time) (*p* = 0.33), nor were there significant differences in lameness scores at trot between weight groups five and three months after FHNE (*p* = 0.64). These results indicate that FHNE provides satisfactory functional outcomes and can be considered a suitable orthopedic intervention for medium- to large-breed dogs.

## Simple Summary

Femoral head and neck excision (FHNE) is a common orthopedic surgical procedure that aims to alleviate pain by reducing bony contact between the femoral head and the acetabular surface, as well as addressing soft tissue damage associated with abnormal hip joint conditions. Previous studies have recommended this technique more often for small- to medium-sized breeds rather than large and giant breeds. Furthermore, it has been shown to yield better outcomes with functional recovery in small- to medium-sized dogs compared to large dogs, as indicated in earlier research. This study aimed to assess hindlimb function after FHNE using data obtained from ground reaction force (GFR) measurements via force plate gait analysis, and other orthopedic evaluations. The results strongly support the use of FHNE as an effective surgical procedure for restoring weight-bearing function, as assessed through GRFs. In general practice, FHNE continues to be used to relieve pain when other treatments fail to improve the dog’s condition.

## 1. Introduction

Total hip replacement (THR) is widely considered as the gold standard treatment for hip-related conditions caused by traumatic incidents [1] or developmental orthopedic disorders such as hip dysplasia, hip osteoarthritis (OA), degenerative joint diseases, and genetic conditions like Legg-Calvé-Perthes disease [2,3,4,5,6]. One study reported adult dogs undergoing unilateral THR achieved functional recovery in the operated limb (OL) comparable to the non-operated limb (NOL) within two months. However, THR requires significant resources and specialized expertise [7]. Therefore, in many regions, an alternative procedure called femoral head and neck excision (FHNE) or ostectomy (FHO) is more commonly performed for these conditions [6,8].

FHNE is a common orthopedic surgical procedure designed to alleviate pain by reducing bony contact between the femoral head and the acetabular surface, as well as addressing soft tissue damage associated with abnormal hip joint conditions. This procedure has been shown to relieve pain and improve the quality of life in dogs [9,10,11].

Many previous studies have recommended this technique for small- to medium-sized breeds rather than large and giant breeds [12,13]. Functional recovery following FHNE has also been shown to have better outcomes in small- to medium-sized dogs compared to large dogs [6,12,14]. Thus, FHNE is generally suggested for dogs weighing less than 20 kg [6,14]. In general practice, this procedure is frequently employed to alleviate pain when other treatments have failed to improve the dog’s condition [13].

Generally, the assessment of the OL function after FHNE relies heavily on subjective evaluations of the dog’s gait and owner-reported questionnaires [15]. Although visual assessment of gait and limb use is often used, its ability to detect subtle changes and details in a dogs’ gait may be limited, leading to potential inaccuracies. Interpretations are heavily influenced by the observer’s experience [16,17,18]. To address these limitations, force plate gait analysis (FP) is used to objectively identify discrepancies in lameness evaluation in dogs [17,19,20].

Use of FPs is a noninvasive, objective, and quantitative evaluation of the ground reaction forces (GRFs), including peak vertical force (PVF) and vertical impulse (VI), which occur between the paw and the ground during the stance phase of a stride [21]. FP-captured GRFs have been extensively utilized to evaluate the efficacy of various medical interventions and to study the impact of weight-bearing on limb function in different conditions [16,22]. However, despite the widespread use of FHNE, no published studies have established specific weight guidelines for this procedure. Therefore, evaluating the outcomes of FHNE using FP analysis in dogs of various sizes and weights would be valuable.

The aim of this study was to assess hindlimb function after FHNE in dogs of a wide weight range by analyzing data obtained from ground reaction force measurements through FP gait analysis and other orthopedic evaluations.

## 2. Materials and Methods

### 2.1. Animals

This study protocol was approved by the Institutional Animal Care and Use Committee of Khon Kaen University, Thailand (license number: IACUC-KKU-30/64). The dogs remained under the care of their owners throughout the study, and all owners were provided with detailed explanations of the study before signing informed consent forms.

The dogs enrolled in the study underwent FHNE at the Veterinary Teaching Hospital (VTH), Faculty of Veterinary Medicine, Khon Kaen University (KKU), Thailand. The data collection was conducted between 2021 and 2023. Routine blood work was taken, and orthopedic examinations were performed. Radiographs were used to confirm the radiographic signs of unilateral hip luxation, femoral head or neck fractures, or severe osteoarthritis on one side. Selection criteria included unilateral FHNE dogs of any breed, sex, or age with a body weight over 10 kg and no systemic disease based on blood profiles. Additionally, dogs had to be capable of trotting across the force plate during their visit after the FHNE without any complication or issues related to their skeleton structure or nervous system throughout the study period.

Ineligible dogs were those with a short strike phase and an inability to trot across the force platform. Other exclusion criteria included unilateral hip luxation due to pelvic fracture, presence of any fracture, and lumbosacral instability. Dogs were also excluded if they had undergone previous orthopedic surgery involving any part of the index limb, experienced complications such as clinically detectable systemic disease or neurological deficits or had an infection during the study period.

The study was designed as a block-randomized, prospective clinical cohort trial in client-owned dogs divided into 2 groups: group 1, consisting of dogs weighing ≤ 20 kg, and group 2, consisting of dogs weighing > 20 kg. Fifteen dogs per group were required.

### 2.2. Surgical Procedure

All dogs were placed for Lactate Ringer’s solution with a cephalic catheter (5 mL/kg/h, Lactate Ringer’s Solution; General Hospital Products, Pathum Thani, Thailand), premedicated with intravenous diazepam, 0.3–0.5 mg kg (Diapine; Atlantic laboratories corporation, Bangkok, Thailand), and intravenous cefazolin (25 mg/kg; Cefaben^®^, L.B.S. Laboratory corporation, Bangkok, Thailand) as antibiotic prophylaxis. Anesthesia was induced with intravenous propofol (1% Profol^TM^, Baxter Pharmaceuticals India Private Limited, Gujarat, India) titrated to effect for endotracheal intubation. General anesthesia was maintained with isoflurane (Isoflurane USP; Piramal Health Care, Bethlehem, PA, USA) in pure oxygen, adjusted for adequate depth. Before the skin incision, intravenous morphine (0.5 mg/kg; morphine sulfate; The Government Pharmaceutical Organization, Bangkok, Thailand) was slowly administered.

The FHNE procedure was performed by a veterinary surgeon with 17 years of clinical surgical experience in orthopedics (S.H.). Using a craniolateral approach, FHNE was performed as described previously [23]. Following the surgery, each dog was administered cephalexin (Cefaben^®^, L.B.S. Laboratory corporation, Bangkok, Thailand) orally at a dose of 25 mg/kg body weight (BW) every 12 h, tramadol hydrochloride (Tramada-100^®^; L.B.S. Laboratory corporation, Samut Prakan, Thailand) orally at 3 mg/kg BW every 12 h, and carprofen (Rimadyl^®^; Zoetis, NE, USA) orally at 4 mg/kg BW every 24 h. Owners were instructed to continue administering these medications to their dogs for a total of seven days.

### 2.3. Study Protocol

The study protocol was written before the start of the study and agreed upon by all investigators. Each dog enrolled in the study visited the VTH for data collection a total of eight times: at 1, 2, 3, 4, 5, 6, 9 and 12 months post-operation. At each time point, the following assessments were conducted by P.T.: a gait observation, a comprehensive orthopedic examination including measurement of hip joint range of motion, ground reaction force measurements of the hindlimbs, and orthopedic assessment scores (OAS) (Table 1).

### 2.4. Outcome Measures

#### 2.4.1. Ground Reaction Force Measurement: Peak Vertical Force (PVF)

**Figure 1:**
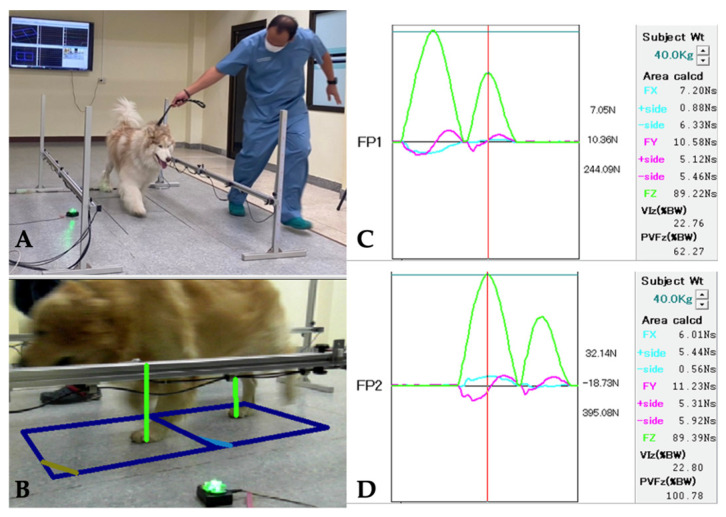
Graphical representation of the GRF measurement (ToMoCo-FPm, Toso System Inc.^®^, Saitama, Japan). (**A**) Each dog was trotted across the FP with velocity controlled within the range of 1.7–2.2 m/s, and acceleration maintained within a range of ±0.5 m/s^2^. (**B**) A video camera recorded each pass to confirm the correct strike of each limb. A valid trial was defined as one in which the forelimb, followed by the ipsilateral hindlimb, struck the force plate. The ground reaction forces (GRFs) are typically represented graphically as peak forces, which consist of three orthogonal directions: mediolateral (Fx), craniocaudal (Fy), and vertical (Fz). The primary data collected in this study were the peak vertical forces (PVF, Fz), which represent the maximum forces generated during the described phase of gait and are depicted by the force–time curve. (**C**) Force plate number 1 (FP1) recorded the ground reaction forces (GRFs) of the right side (forelimb and hindlimb) that occur between the paw and the ground during the stance phase of a stride, along with the pressure distributions. The red line was a marker that could be moved to measure the PVF of each limb (**D**), Force plate number 2 (FP2) represented GRFs of the left side.

PVF was determined using FP gait analysis. This analysis was performed using dual biomechanical strain gauge force plates (Advanced Mechanical Technology^®^, AMTI Model OR6-6, Watertown, MA, USA) 40 × 60 cm in size, each embedded in the middle of an 8 m-long walkway. The dogs were trotted across the FP by the same handler (P.T.). Signals from the dual force plates were collected and processed using dedicated gait analysis software (ToMoCo-FPm, version 12.02, Toso System Inc.^®^, Saitama, Japan) to retrieve PVF values. Velocity was measured using four laser sensors (photocells) mounted 50 cm apart on each side of the walkway. Velocity was maintained between 1.7–2.2 m/s, with acceleration maintained within a range of 0.5 m/s^2^ throughout the study. A video camera (Panasonic HC-V180, Panasonic, Osaka, Japan) recorded each pass to confirm the correct strike of each limb. A valid trial was defined as the forelimb, followed by the ipsilateral hindlimb, striking the force plate. The initial PVF value was recorded in Newton meter (Nm) normalized to body weight and expressed as a percentage of total body weight (%BW) for each limb. The mean PVF value for each evaluation time point was calculated from the average of the three valid trials. Because the dogs were unable to bear weight on the affected limb at the first visit, the PVF value of the OL one month post-operation was used as a baseline to evaluate improvements in limb function throughout the study period, as shown in Figure 1. Symmetry indices (SI) were calculated from the PVF values of the hindlimb for each dog using the previously described formula: SI = [(mean NOL − mean OL)/0.5 × (mean NOL + mean OL)] × 100 [25]. The SI results were compared between the OL and NOL at each evaluation time point.

#### 2.4.2. Hip Joint Range of Motion

The range of motion of both hip joints was measured using a standard transparent plastic goniometer [26]. Dogs were positioned in lateral recumbency, ensuring a neutral and relaxed position for the measurements. The goniometer measurement technique followed previously described methods [26,27]. The total range of motion in the hip joint was determined by assessing the position and angle of flexion and extension, with limitations imposed by pain, discomfort, or mechanical tissue-related restrictions. The NOL served as a control.

#### 2.4.3. Orthopedic Assessment Scores (OAS)

The OL function was assessed using the subjective gait analysis at each time point, following the subjective orthopedic grading system known as OAS. Lameness at trot was evaluated during trotting, and scores were assigned according to Table 1. This study adapted the system from [24].

The focus on lameness evaluation during trotting was chosen because this gait is commonly used for assessing symmetry in weight-bearing across all four limbs in quadrupeds. Although not formally defined or tested, a category change to a score of ‘0’ is considered clinically relevant.

### 2.5. Statistical Analyses

To explore continuous variables at the first visit including age, BW, kinetic data (PVF and SI), and hip range of motion, an independent t-test was used across groups. The OAS was analyzed using the Mann–Whitney U test. The PVF of OL was used to calculate the changes in PVF at each time point after one month post-operation. The results, including PVF of OL, changes in PVF of OL, and SI between weight groups, were tested using a linear mixed model for repeated measurement to explore effect of group (BW ≤ 20 kg and >20 kg), and time over one year follow-up, as well as their interaction (STATA v10.1, University licensed, StataCorp LLC, College Station, TX, USA). The main factors (fixed effects) were treatment group, visit and their interaction, while the random factor was the subject’s response measured at multiple time points with the variance component as unstructured. To explore the simple effect, multiple comparisons between treatment groups at each time point were performed by post-estimation command (CONTRAST) with Bonferroni method to correct the *p*-value. Additionally, a linear mixed model with repeated measurement was used to test the effect of group on the OAS outcomes.

The period from the time after surgery until the PVF value reached a level that was equal to or less than 5% of the PVF value in the NOL was considered as the functional recovery time. The recovery time data (in months) were compared between groups (≤20 kg and >20 kg) by survival analysis over the 12-month observation period following surgery. Additionally, another method of evaluating recovery time involved assessing the time until the lameness score at trot reached 0. Cases that recovered during the observation period were considered as uncensored time, while cases that did not recover during this time were considered censored. Survival analysis was used to estimate the Kaplan–Meier curve and compare the survival data using the log-rank test. The significance level was set at a *p*-value < 0.05 for all statistical calculations.

## 3. Results

Initially, 30 FHNE dogs were enrolled and divided into two groups as follows: 15 dogs in the group with a body weight ≤ 20 kg, and 15 dogs in the group with a body weight > 20 kg. However, during the study, 3 dogs in the group with a body weight ≤ 20 kg dropped out due to loss of contact (*n* = 1) and repeated accidents (*n* = 2). Therefore, 27 dogs (Table 2) were used in the study. Of these, 15 were males and 12 were females, with ages at the time of surgery ranging from 6 months to 10 years. The average (mean ± SD) age, body weight (BW), and body condition score (BCS) were 3.22 ± 3.05 years, 23.34 ± 8.04 kg (ranging from 11.55 to 38.78 kg), and 3.04 ± 0.65, respectively. Ten breeds of dogs participated, including mixed breeds (*n* = 10), Poodle (*n* = 1), Spitz (*n* = 1), Golden retriever (*n* = 5), Labrador retriever (*n* = 5), Alaskan malamute (*n* = 1), Chao Chao (*n* = 1), Samoyed (*n* = 1), Siberian husky (*n* = 1), and Thai ridgeback (*n* = 1). In the group with a body weight ≤ 20 kg, mixed breeds were the predominant breed, accounting for 10 dogs (37.04% of the total). In the group with a body weight > 20 kg, Golden and Labrador retriever were the predominant breed, with each accounting for 5 dogs (18.52% of the total).

In this study, the index limb was the right hindlimb in 14 dogs and the left hindlimb in 13 dogs. The most common reason for surgery was vehicular trauma (luxated hip joint), accounting for 16 cases (59.26% of the total). Secondary etiological causes included hip disease, dog fights, falls from heights, limb pulling, and slips and falls. Overall, 21 dogs had hip luxation as the primary diagnosis, while the remaining cases were diagnosed with a femoral head or neck fracture (*n* = 4) or hip OA with associated pain (*n* = 2).

The contralateral limbs which were not operated on served as ‘controls’. A total of 15 dogs had normal hips on the contralateral side, while others showed signs of hip dysplasia or varying degrees of hip OA, ranging from early-stage OA to severe hip OA, as presented in Table 2. The characteristics of the dogs, including age, BW, BCS, kinetic data (PVF and SI), hip flexion or hip extension, and OAS were recorded as baseline measurements at one month post-operation. These are presented in Table 3. No significant differences were observed between the body weight groups (*p* > 0.05).

### 3.1. Force Plate Gait Analysis: Peak Vertical Force (PVF)

**Figure 2:**
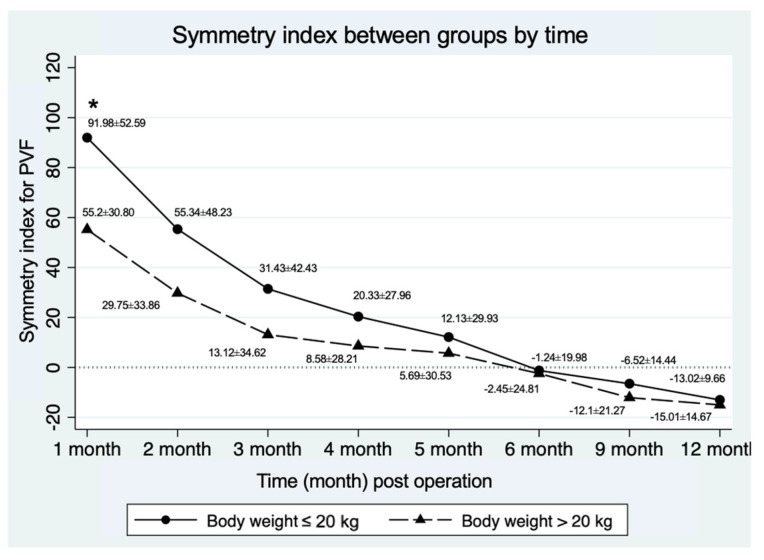
Graphical representation of the SI (mean ± SD) between groups (≤20 kg and >20 kg) during the study period. * Indicate a significant difference.

The PVF expressed as a percentage of body weight for the OL showed no significant differences between groups at any follow-up time point. The mean change from baseline (one month post-operation) in PVF expressed as a percentage of body weight for the OL was not different between groups (*p* = 0.71) at any time during the follow-up period, as presented in Table 4. However, SI for PVF showed a significant difference at one month post-operation, as shown in Figure 2, with SI being greater (less symmetrical use of the hindlimbs) in the group that was ≤20 kg.

**Figure 3:**
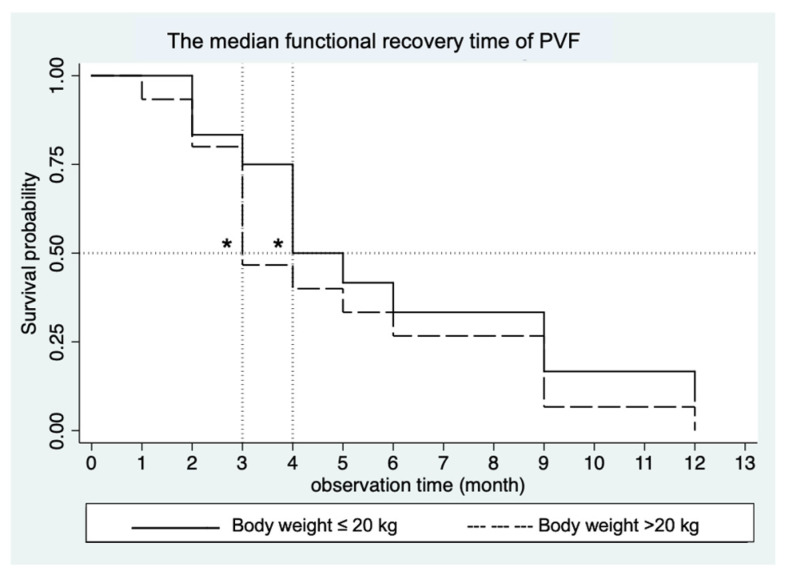
Graphical representation of the median functional recovery time of PVF between groups (≤20 kg and >20 kg). The median time was 4 months for dogs with a BW ≤ 20 kg and 3 months for dogs with a BW > 20 kg after surgery. * Indicate median recovery times estimated by 0.5 survival probability.

The mean SI showed significant differences between groups only at one month post-operatively. At six months post-operatively, the SI value was close to zero, indicating that OL weight-bearing was the same as NOL. The median functional recovery time (in months) was compared between the groups (≤20 kg and >20 kg) using survival analysis over the 12-months observation period following surgery. Among the dogs, 11 out of 12 dogs (91.67%) in the BW ≤ 20 kg group and 15 out of 15 dogs (100%) in the BW > 20 kg group exhibited a normal gait within 12 months (*p* = 0.44)—with normal gait being defined as a difference in PVF values between the OL and the NOL, ≤5%BW at the specified time point was considered indicative of recovery. Dogs in the ≤20 kg group showed no significant difference in the median functional recovery time compared to those in the >20 kg group (*p* = 0.33) at 4 months (95% CI; 2.9) and 3 months (95% CI; 2.6), respectively, as shown in Figure 3.

### 3.2. Orthopedic Assessment Scores (OAS)

**Figure 4:**
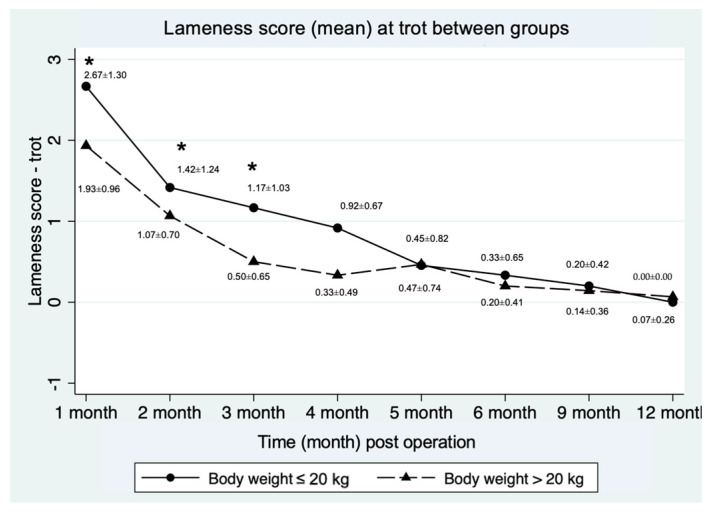
Graphical representation of the mean lameness score at the trot between groups (≤20 kg and >20 kg). * Indicates that the time point was significantly different between groups.

The mean lameness score at trot between groups showed significant differences at month 1, 3 and 4 post-operation, as shown in Figure 4.

**Figure 5:**
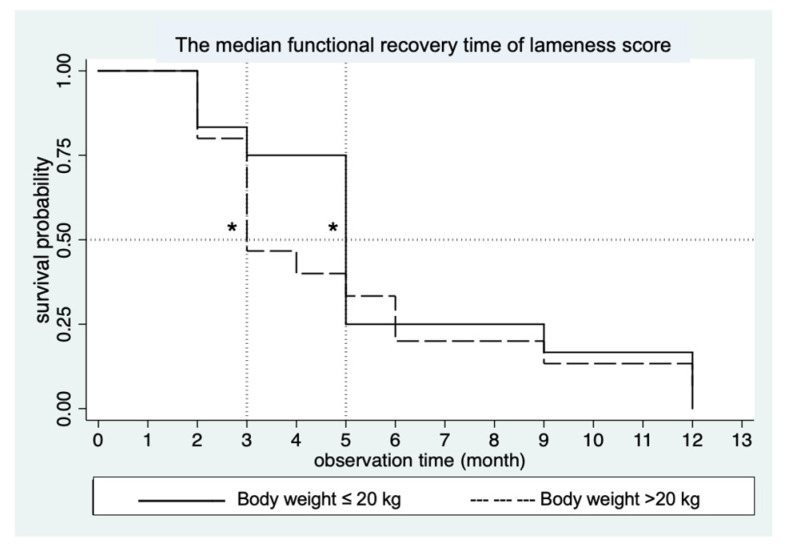
Graphical representation of the median functional recovery time of lameness score. The median time was at 5 months for dogs with a BW ≤ 20 kg and 3 months for dogs with a BW > 20 kg post-operation. * Indicate median recovery times estimate by 0.5 survival probability.

The lameness score of 0 was observed and considered as full recovery. Recovery time data (in months) were compared between the groups (≤20 kg and >20 kg) using survival analysis within a 12-months observation period post-operation. In the BW ≤ 20 kg group, all 12 dogs (100%) exhibited normal gait within 12 months, while in the >20 kg group, 14 out of 15 dogs (93.33%) achieved normal gait (*p* = 0.55). There was no significant difference in recovery time between the ≤20 kg group and the >20 kg group (*p* = 0.64) at 5 months (95% CI; 2.9) and 3 months (95% CI; 2.6), respectively as shown in Figure 5.

### 3.3. The Range of Motion (ROM) of Hip Joint Flexion and Extension

All dogs that underwent FHNE in each group during the study period showed no significant difference in ROM between the two groups (*p* > 0.05) for any criteria. The overall outcomes of hip joint angle at flexion for dogs with a BW > 20 kg and those with a BW ≤ 20 kg were within the normal range, comparable to the NOL at 5 and 9 months after FHNE, respectively. Meanwhile, the overall outcomes of hip joint angle in extension for the OL in both groups were slightly closer to the values of the NOL and remained within the normal range at 12 months post-operation. When comparing the ROM between the groups, no significant difference was observed at the 12-month mark (Table 5).

## 4. Discussion

This study indicates that FHNE is an effective orthopedic procedure which provides satisfactory functional outcomes and can be performed in medium- to large-sized dogs. The results showed that GRFs, as measured by PVF and lameness score at trot, did not differ between the OL and NOL in dogs weighing ≤ 20 kg (11.55 to ≤20 kg) or in those weighing > 20 kg (>20 to 38.78 kg) with a median functional recovery time of 4 months and 3 months for GRFs, and 5 months and 3 months for lameness score, respectively.

GRFs serve as objective measures and have been utilized as a proxy estimate of joint pain in dogs with appendicular joint OA. They are widely employed to evaluate the effectiveness of various medical interventions and to study the outcome of weight-bearing on limb function in various conditions [16,22,28,29,30,31,32,33]. The SI gives an indication of the symmetry of limb use by comparing the index limb to the contralateral limb, determining whether perfect symmetry or a deviation exists [34]. Manley et al. [7] reported on a study involving 10 adult dogs, weighing between 30 and 37 kg, that underwent unilateral THR. They evaluated the functional outcomes using GRFs after the surgery and found that, at 2 months post-operation, the functional recovery of the OL was not different from that of the NOL. In a study using ground GRFs to evaluate PVF at a walk after FHNE, it was found that on day 120, the PVF at a walk in dogs that underwent FHNE was not significantly different from the values obtained for control dogs [35].

To our knowledge, no published studies have evaluated GRFs at trot in FHNE-treated dogs of various sizes and weights through a series of time-interval measurements after surgery. In this study, the GRFs were measured using force plates at 1, 2, 3, 4, 5, 6, 9 and 12 months after operation. The time intervals between these measurement points were not uniform. During the first 6 months, evaluations were conducted monthly, as functional recovery was anticipated within this period. After the initial 6 months, evaluations were conducted at three-month intervals. Functional recovery time in this study was defined as the point at which the SI showed no difference or a difference of less than 5%BW.

Several previous studies have recommended performing FHNE in small- to medium-sized breeds of dogs rather than in large and giant breeds [12,13]. Functional recovery time after FHNE has been shown to have better outcomes in small- to medium-sized dogs compared to large dogs [6,12,13,14]. Additionally, it is also suggested in other studies [6,14] that FHNE be performed in dogs weighing less than 20 kg. In contrast, some studies have indicated that large breeds may experience a shorter recovery time than smaller dogs for FHNE [6,36,37]. Our study found good results for FHNE, suggesting that it can be performed in dogs of varying sizes (ranging from 11.55 to 38.78 kg in this study). In this study, we observed no significant difference in weight-bearing between the OL and the NOL in both groups 1 (11.55 to ≤20 kg) and 2 (>20 to 38.78 kg), with a median functional recovery time of 4 and 3 months (95% CI) post-operation, respectively. Large dogs tended to have a shorter recovery time compared to small dogs; however, this shorter recovery time observed in group 2 did not reach a statistically significant (*p* = 0.33). It is noteworthy that the recovery time for the studied dogs varied within a range of 1 to 12 months. Our findings regarding outcomes in large dogs (group 2, >20 kg) differed from previous reports suggesting that small- to medium-sized breeds recover normal limb function faster than large breeds [6,12,13].

Several factors can contribute to variations in the recovery time post-operation, including those related to the FHNE procedure. These factors include the dog’s age, breed, behavior, activity level, lifestyle, management by the dog owner, duration of the condition before surgery, surgical technique, and the orthopedic surgeons’ skill [13,36]. In this study, we minimized the impact of the surgeon factor by utilizing a single orthopedic surgeon for all dogs. Previously published reports of clinical trials have suggested that younger dogs generally recover more quickly and achieve better outcomes than older dogs following FHNE [6,13,35,36,37]. In our study, the majority of dogs were young, which could potentially affect the recovery time. Another factor that may have influenced the GRFs in this study was the underlying conditions of the NOL. In group 2, 12 out of 15 dogs (80%) had OA, which could contribute to variation in GRFs. The presence of hip OA can cause pain, leading to a decrease in the GRFs value or a shift in weight-bearing. We observed reduced weight-bearing on the NOL, as indicated by the GRFs, in some dogs, particularly in group 2, several months post-operation. This finding may be attributed to the worsening of OA in the NOL, resulting in decreased GRFs of the NOL. If the contralateral limb is not being used much, then symmetry suggests that the OL may perform as poorly as the contralateral limb.

Variations in GRFs during force plate gait analysis can be influenced by several factors. Controlling velocity is essential to limit gait data variability; therefore, in our study, we maintained a velocity range of 1.7–2.2 m/s and an acceleration range of 0.5 m/s^2^. Our study’s dog weights varied from 11.55 to 38.78 kg. Subject size also affects GRFs, which we minimized by normalizing data to body weight, expressed as a percentage (%BW). This normalization allows for comparison between dogs of different sizes and accounts for individual weight differences over time. Additionally, using different handlers can introduce up to 7% variance in kinetic data [38]. To reduce this variability, we employed a single handler for all gait collections to lead the dogs across the force plates [30].

Orthopedic examination was performed in all dogs by a single veterinarian during each visit to minimize variation. The interpretation of lameness can be challenging to assess subjectively due to the difficulty in visually observing subtle changes and details in a dog’s gait characteristics, leading to variability. Subtle lameness may not be apparent during subjective gait evaluation and can be difficult to detect [39]. Several studies have shown that subjective evaluation of lameness in dogs may or may not correlate with the presence or absence of severe lameness, indicating low reliability in detecting lameness [18,19]. However, in this study, the lameness evaluation based on OAS (*p* = 0.64) correlated with GRFs (*p* = 0.33), an objective evaluation. The lameness score showed no difference between the OL and the NOL in both groups 1 and 2, with a median functional recovery time of 5 and 3 months (95% CI) after surgery, respectively. This finding aligns with the GRFs results. The overall of the OAS’s outcome was not significantly different among groups in our study.

Engstig et al. [9] found that lameness at walk and trot persisted in dogs with low-grade conditions after FHNE, with poor outcomes associated with lameness lasting over six months, while excellent results correlated with shorter durations [6,35] of observed lameness in 56% of dogs post-FHNE, and Charette et al. [40] noted mild pain due to overextension in some dogs. These assessments were based on owner evaluations and the OAS. Clinical metrology instruments (CMIs) involve caregiver observations and can validly measure OA pain’s impact if culturally and linguistically relevant. However, a study in Thailand [41] showed that a translated CBPI was not fully understood. Consequently, no CMIs were used in this study due to a lack of validation in the Thai language and culture [22].

In this study, the ROM of both hip joints was determined using a standard transparent plastic goniometer, as previously described [26]. The overall outcomes of hip joint flexion values in the OL of group 1 and group 2 were within the normal range, similar to the NOL, at 9 and 5 months after the surgery, respectively, as previously reported [26]. Likewise, the overall outcomes for hip joint extension in the OL of both groups were slightly closer to the values of the NOL and within the normal range, consistent with previous findings at 12 months after surgery [26]. When comparing the ROM between group 1 and group 2, no significant differences were observed at the 12-month mark.

The results for the angle of the operated hip joint during extension in this study differed from previous findings, which reported a decreased angle after FHNE [9,13,35]. Engstig et al. [9] found that eight out of ten dogs exhibited reduced extension in the OL at a mean of 2.7 years after the operation [9], while another study reported that 74% of 66 dogs had restricted hip movement at a mean of 4 years after FHNE, while flexion was generally unaffected [6]. The limited hip extension observed in FHNE cases is also common in hip dysplasia and hip OA. In fact, it has been suggested that a reduction in joint motion of less than 10 degrees is unlikely to significantly impact limb function, whereas more severe restrictions may affect the dog’s gait [42]. The decrease in the hip joint angle observed in previous studies was likely due to the formation of a fibrous pseudoarthrosis, which restricted movement and did not improve over time in the OL. The development of fibrous ankylosis in the pseudoarthrosis might also result from shortening of the OL, which in turn affected the related trot [35].

Limitations of this study include its small sample size, which may affect the results, and the lack of direct comparison to either similar research or the THR technique. Future research should utilize larger cohorts, conduct long-term follow-ups or studies to monitor and compare different surgical techniques such as THR, as well as various rehabilitation protocols aimed at reducing recovery time and facilitating a return to normal function after FHNE.

## 5. Conclusions

Our study indicates that the FHNE provides satisfactory functional outcomes for restoring symmetrical weight-bearing in the hindlimbs of dogs. In general practice, FHNE can be considered a suitable orthopedic procedure for medium- to large-breed dogs. Although the recovery time for hip function may be longer with FHNE than with THR, the results suggest that a limb treated with FHNE can achieve function comparable to the non-operated limb (median functional recovery time) within four months, regardless of the dog’s size or weight.

## Figures and Tables

**Table 1:** Grading of orthopedic assessment scores (OAS) for lameness evaluation at trot [24].

Criterion	Clinical Evaluation
Lameness at trot	0. No lameness/weight-bearing on all strike observed 1. Mild subtle lameness with partial weight-bearing 2. Obvious lameness with partial weight-bearing 3. Obvious lameness with intermittent weight-bearing 4. Full non weight-bearing

**Table 2:** Subject characteristics and baseline data for dogs in each body weight group prior to surgery.

Variable	BW ≤ 20 kg n = 12	BW > 20 kg n = 15	Total n (%)
Sex			
Female	6	6	12 (44.44)
Male	6	9	15 (55.56)
BCS			
2	2	3	5 (18.52)
3	7	9	16 (59.26)
4	3	3	6 (22.22)
Breed			
Mixed breed	10	0	10 (37.04)
Poodle	1	0	1 (3.70)
Spitz	1	0	1 (3.70)
Golden retriever	0	5	5 (18.52)
Labrador retriever	0	5	5 (18.52)
Alaskan malamute	0	1	1 (3.70)
Chao Chao	0	1	1 (3.70)
Samoyed	0	1	1 (3.70)
Siberian husky	0	1	1 (3.70)
Thai ridgeback	0	1	1 (3.70)
Side of affected limb			
Right	8	6	14 (51.85)
Left	4	9	13 (48.15)
Etiology of case			
Vehicular trauma	11	6	16 (59.26)
Falls from heights	1	0	1 (3.70)
Hip disease (OA, HD)	0	5	5 (18.52)
Dog fights	0	3	3 (11.12)
Pulled the limb	0	1	1 (3.70)
Slips and falls	0	1	1 (3.70)
Radiographic finding (operated limb)			
Craniodorsal dislocation	10	11	21 (77.78)
Femoral head fracture	1	2	3 (11.12)
Neck of femoral head fracture	1	0	1 (3.70)
Progressive hip OA	0	2	2 (7.40)

**Table 3:** Baseline information of the operated limb (OL) in dogs of different body weight groups at one month post-operation.

Variable (Presented as Mean ± SD)	BW ≤ 20 kg n = 12	BW > 20 kg n = 15	*p*-Value
PVF **	31.16 ± 16.52	33.25 ± 10.71	0.71
Symmetry index for PVF **	91.98 ± 52.59	55.20 ± 30.80	0.04
Hip flexion **	45.83 ± 6.69	44.67 ± 8.96	0.71
Hip extension **	138.33 ± 17.10	145.00 ± 13.76	0.27
OAS ***			
Lameness score at trot	2.67 ± 1.30	1.93 ± 0.96	0.10

OAS, Orthopedic assessment scores. ** independent *t*-test. *** Mann–Whitney U test.

**Table 4:** PVF values at one month post-operation and the mean changes in PVF (±SD) in the OL for each group at 2, 3, 4, 5, 6, 9, and 12 months following FHNE surgery. * Indicates that the mean change in PVF of the index limb was significantly different (*p* < 0.05) from that at Month 1 within the same group.

Visited Time (mean ± SD)	PVF			Mean Change PVF		
	BW ≤ 20 kg	BW > 20 kg	*p*-Value	BW ≤ 20 kg	BW > 20 kg	*p*-Value
	n = 12	n = 15		n = 12	n = 15	
1 month post-operation	31.16 ± 16.52	33.25 ± 10.71	0.76	-	-	-
Month 2	44.69 ± 19.13 *	48.28 ± 13.07 *	0.86	13.71 ± 9.39	14.78 ± 8.10	0.98
Month 3	53.28 ± 18.51 *	52.93 ± 17.87 *	0.88	22.12 ± 12.77	18.77 ± 11.25	0.43
Month 4	56.37 ± 12.02 *	58.00 ± 15.86 *	0.88	27.57 ± 12.93	23.73 ± 11.50	0.37
Month 5	62.12 ± 16.54 *	56.19 ± 18.52 *	0.33	30.96 ± 13.70	21.83 ± 13.88	0.06
Month 6	67.31 ± 14.34 *	62.62 ± 17.71 *	0.44	36.15 ± 13.98	28.79 ± 13.44	0.15
Month 9	68.67 ± 8.92 *	66.68 ± 17.5 *	0.40	41.045 ± 13.81	33.08 ± 11.50	0.09
Month 12	73.43 ± 10.61 *	71.86 ± 15.95 *	0.77	42.51 ± 16.52	37.19 ± 10.65	0.34

**Table 5:** ROM (mean ± SD) of hip joint flexion and extension in the hindlimb for both OL and NOL in each group at 1, 2, 3, 4, 5, 6, 9, and 12 months following FHNE surgery.

Visit Time	Hip Flexion				Hip Extension			
	BW ≤ 20 kg n = 12		BW > 20 kg n = 15		BW ≤ 20 kg n = 12		BW > 20 kg n = 15	
	OL	NOL	OL	NOL	OL	NOL	OL	NOL
Month 1	45.83 ± 6.69	47.92 ± 6.56	44.67 ± 8.96	49.33 ± 7.99	138.33 ± 17.10	159.17 ± 14.90	145.00 ± 13.76	157.67 ± 11.16
Month 2	45.83 ± 7.02	49.17 ± 7.02	47.00 ± 8.82	50.00 ± 6.27	140.00 ± 17.58	165.00 ± 11.28	149.67 ± 16.20	161.33 ± 9.54
Month 3	48.75 ± 4.83	49.58 ± 4.50	48.21 ± 8.23	49.64 ± 7.96	152.08 ± 14.99	166.67 ± 6.15	154.64 ± 11.84	162.86 ± 8.48
Month 4	45.91 ± 5.84	50.45 ± 4.72	49.67 ± 7.43	51.33 ± 8.34	153.64 ± 14.51	170.00 ± 9.22	156.00 ± 9.86	164.33 ± 8.21
Month 5	50.00 ± 5.22	51.67 ± 6.85	53.00 ± 5.28	51.00 ± 7.37	157.5 ± 14.69	169.17 ± 5.57	158.00 ± 10.66	162.67 ± 9.42
Month 6	50.83 ± 4.17	51.25 ± 5.28	52.67 ± 7.04	53.00 ± 6.49	159.58 ± 9.16	167.50 ± 3.99	157.33 ± 9.42	160.00 ± 9.06
Month 9	52.50 ± 2.64	51.50 ± 5.30	52.86 ± 5.08	55.00 ± 3.40	159.50 ± 6.85	168.50 ± 3.37	157.86 ± 8.93	160.00 ± 9.20
Month 12	54.55 ± 2.70	55.00 ± 3.16	55.33 ± 3.99	55.67 ± 5.30	160.00 ± 9.75	165.00 ± 4.47	159.00 ± 5.73	161.67 ± 5.23

## Data Availability

All data is contained within this article.

## Abbreviations

The following abbreviations are used in this manuscript:

BCS	Body condition score
BW	Body weight
CBPI	Canine Brief Pain Inventory
CMIs	Clinical metrology instruments
FP	Force plate gait analysis
FHNE	Femoral head and neck excision
FHO	Femoral head and neck osteotomy
GRFs	Ground reaction force measurements
HD	Hip dysplasia
NOL	Non-operated limb
OL	Operated limb
OAS	Orthopedic assessment scores
OA	Osteoarthritis
PVF	Peak vertical force
ROM	Range of motion
SI	Symmetry indices
THR	Total hip replacement
VTH	Veterinary Teaching Hospital
VI	Vertical impulse
